# Synthesis of an Al^18^F radiofluorinated GLU-UREA-LYS(AHX)-HBED-CC PSMA ligand in an automated synthesis platform

**DOI:** 10.1186/s41181-018-0039-y

**Published:** 2018-02-27

**Authors:** Javier Giglio, Maia Zeni, Eduardo Savio, Henry Engler

**Affiliations:** Uruguayan Center of Molecular Imaging (CUDIM), Montevideo, Uruguay

**Keywords:** [^18^F]AlF-PSMA, Automated synthesis, ^68^Ga-PSMA, Tracerlab FXFN® (GE) platform

## Abstract

**Background:**

Overexpression of prostatic membrane antigen (PSMA) is associated with the progression and prognosis of prostate cancer. There are numerous studies using this peptide with the ^68^Ga radionuclide. Previous methods to synthetize ^18^F–labeled PSMA ligands with complexes [^18^F]AlF^2+^ have been achieved. However, these reported syntheses were performed manually, using small volumes. Therefore it is only possible to have the radiopharmaceutical on a small scale, for use in preclinical studies. ^18^F–labelled tracers allow higher doses increasing the number of examined patients. In addition, late images can be acquired in the case of uptake in lymph nodes, to discard inflammation. It is important to transfer the manual synthesis to an automatic module, producing a batch of the radiopharmaceutical with high activity in a safe and effective way. The aim of this work was to optimize the labeling of [^18^F]AlF-[GLU-UREA-LYS(AHX)-HBED-CC] in a Tracerlab FXFN® (GE) platform.

**Results:**

The labeling up to the reactor corroborates the formation of the complex [^18^F]AlF-PSMA. After purification by HPLC, the radiopharmaceutical was achieved with a radiochemical purity higher than 90%. The quality control of the final product fulfilled all the requirements in agreement with USP, such as radiochemical purity (greater than 90%) and residual solvents. [^18^F]AlF-PSMA was obtained with a yield of 18 ± 3% (*n* = 7), not decay corrected (NCD) starting off from 500 to 2000 mCi the 18F and with a radiochemical purity of 95 ± 3% (n = 7). The product verified stability in the final formulation vial during 4 hs and in human plasma up to 1 h.

**Conclusion:**

The proposed method allowed the production of [^18^F]AlF-PSMA with suitable radiochemical purity in a commercial platform. High activities were achieved, with a simple and robust methodology appropriate for clinical purposes.

## Background

The molecular imaging of positron emitters (PET) provides a sensitive and specific technique for the “in vivo” evaluation of biological processes.

Overexpression of prostatic membrane antigen (PSMA) is associated with the progression and prognosis of prostate cancer. Among all the markers studied, such as antibodies, aptamers and small molecules, the one that has had more relevance in the clinical use is the PSMA. There are numerous studies using this peptide with the ^68^Ga radionuclide (Ghosh and Heston 2004).

The most widely used radionuclide is the ^18^F isotope, being a low energy emitter (β + 0.635 MeV (97%)) and having a suitable half-life of 109.8 min. So this radionuclide presents the ideal properties to be used in molecular imaging and also provides the best spatial resolution of around 2 mm (Peter et al. 2014). Usually ^18^F is covalently bonded to molecules, to form the radiopharmaceutical (McBride et al. 2013a).

Typically the introductions of ^18^F require elevated temperature and organic solvent as reaction conditions, which are often incompatible with peptide stability. To overcome this limitation, the classical labeling method is usually employed using N-succinimidyl-4- [^18^F] flourobenzoate (SFB) or the use of [^18^F] fluorobenzaldehyde to react with a aminooxy or hydrazino nicotinamide derivatized peptide (Poethko et al. 2004; Rennen et al. 2007; Bruus-Jensen et al. 2006). As another alternative the “click chemistry” reactions have been studied for labeling of different peptides (Iddon et al. 2011). This pathway requires the use of multi-step protocol that make more cumbersome and with lower yields than direct labeling.

It is possible to combine the rich chemistry of labeled peptides with a radionuclide that provides excellent decay characteristics such as ^18^F (Da Pieve et al. 2016; Meyer et al. 2016), using the Al^18^F complex in the peptide labeling. This mehodogy has recently been of great interest, since it would allow direct synthesis of the labeled peptide in a few steps (McBride et al. 2009; 2013b; Chang et al. 2016).

These methods have been successfully employed in the synthesis of different peptides and small proteins. Yu et al. described the use of this technique in the labeling of the F-NOTA-E [PEG4-c (RGDfk)] 2 (denoted as [^18^F] Alfatide II) in 9 patients with cerebral metastasis and in 5 healthy volunteers. They demonstrated the clinical usefulness of this type of labeling using the Al^18^F^+ 2^ complexes (Yu et al. 2015).

Previous methods to synthetize ^18^F–labeled PSMA ligands with complexes [Al^18^F]^2+^ have been achieved (Boschi et al. 2016). However, these reported syntheses were performed manually, using small volumes. Therefore it is only possible to have the radiopharmaceutical on a small scale, for use in preclinical studies.

However, the work of S. Lütje, et al. presented in the EANM 2017, obtain a product which is not stable in PBS, TFA or ethanol. In addition, the product presented in vivo a bone uptake associated with instability of the compound. (Lütje et al. 2017). Nevertheless E. Al-Momani et al. (Al-Momami et al. 2017) reported that the stability of the radiopharmaceutical was highly dependent of the formulation conditions, being stable in saline solution with 1% of ethanol or acetate buffer pH 6, and not stable in ethanol 10%/PBS.

The aim of this study is to establish a synthesis methodology an automated platform in order to achieve high activities. This would to perform a larger number of patients to be accomplished than with the ^68^Ga labeling.

## Methods

### Reagents

Chemicals: Glu-urea-Lys (Ahx)-HBED-CC (PSMA-11) was purchased from ABX (Radeberg, Germany). AlCl_3_.6H_2_O, sodium acetate trihydrate and acetic acid were all metal free. Sodium phosphate, ethanol, water, acetonitrile and trifluoroacetic acid (TFA) were of high-grade purity. Sep-Pak Accell Plus QMA cartridge were from Waters. ^18^F was obtained in a PETtrace cyclotron (16.5 MeV, GE Healthcare) using a 2.5 mL Niobium target via ^18^O (p,n) ^18^F reaction. Preparation of ^18^F–fluoride: QMA cartridge was conditioned with 5 mL of 0,5 M sodium acetate, followed by 10 mL of water (Milli Q, 18,5 MΩ). Activity measurements were performed with a dose calibrator Capintec CRC 25R, CRC 25 PET or a 3”× 3” well type NaI(Tl) solid scintillation detector coupled to a multichannel analyzer ORTEC.

Preparation of solution: Sodium acetate/acetic acid buffer 0,5 M and 0,05 M (pH 4,5) were prepared from each component and mixing them to obtain the desired final pH. PSMA-11 was dissolved in water (2 mg/mL) and aliquots were frozen stored (− 18 °C). AlCl_3_.6H_2_O 0,01 M was prepared in 0,05 M acetate buffer (pH 4) and stored at 4 °C.

#### ^18^F–radiolabeling conditions using GE TRACERlab FX-FN automated synthesis platform

**Fig. 1:**
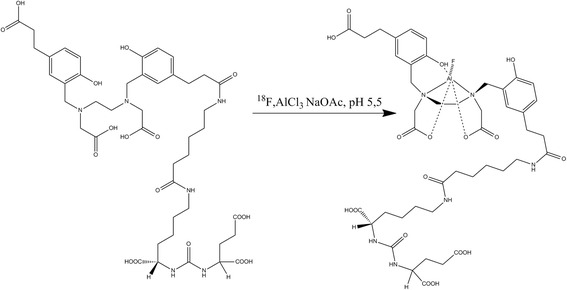
Synthesis scheme with the proposed structure

This study to describe the development of a [^18^F]AlF automated radiolabelling procedure. The reaction is carried out according to the scheme shown in Fig. 1.

**Fig. 2:**
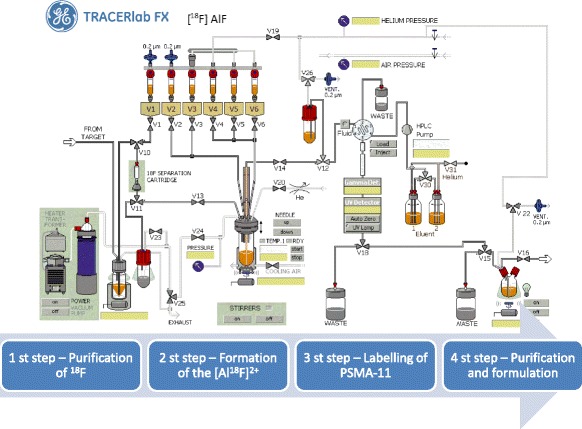
Upper: schematic representation of the [^18^F]AlF-PSMA-11 radiochemistry, lower: diagram of synthesis steps of [^18^F]AlF-PSMA-11

The synthesis was carried out on a commercial Ge TRACERlab FXFN platform according to the steps shown in Fig. 2, using the V15 valve to perform HPLC peak collection direct the product vial. The labeling process was carried out in 4 stages, as follows:

#### First step

The [^18^F] fluoride was received in the module and purified by a Sep-Pak ligth QMA, placed between position V10 and V11. The ultrapure water was sent from the target to wash the QMA column.

#### Second step

[^18^F] fluoride is eluted from the QMA into the reactor via vial 1 with 500 μL of 0.5 M sodium acetate buffer (pH 4.5). The reactor contains 4.5 μL of AlCl_3_ in 0.5 M acetate buffer (pH 4.5). The formation of the [Al^18^F]^+ 2^ complexes was carried out for 5 min at room temperature.

#### Third step

Subsequently to the synthesis of the complex [Al18F]+ 2, a solution containing 500 μL EtOH, 700 μL 0.5 M NaOAc and 30 μL PSMA-11 (2 μg/μL) was added from vial 4, which was them heated for 10 min at 50 °C.

#### Fourth step

The reaction mixture was diluted with 3 mL of 10 mM buffer Phosphate (pH 6.8), prior to its injection into the HPLC system from vial 6. Purification was performed in a preparative M-N VP250/16 Nucleosil 100-5C18 column using two mobile phases. The first mobile phase was for the elution of free ^18^F^−^ and [Al^18^F]^+ 2^, and the second one was for the elution of the product (see Table 1), using UV at 220 nm and gamma detection.

**Table 1:** Conditions of HPLC for purification

Time (min)	Mobile phase	Flow (mL/min)
0–15	phosphate Buffer 10 mM pH = 6.8: EtOH (98:2)	8
15–20	phosphate Buffer 10 mM pH = 6.8: EtOH (92:8)	8
20–25	phosphate Buffer 10 mM pH = 6.8: EtOH (92:8)	4

The peak of the radiopharmaceutical was collected directly into the product vial and transferred to the sterile vial by sterilizing filtration using a Millex GV 33 mm 0.22 um filter.

### Quality control

The radiochemical purity (RP) was determined using HPLC analysis, a reverse phase column Agilent ZORBAX Eclipse Plus C8 4.6x150mm 5 μm column, flux 2.0 mL/min and the following solvent gradient: (A) TFA 0.1% (*v*/v) in water, (B) TFA 0.1% (v/v) in acetonitrile; 0–7 min 0 %B, 7–10 min (0–50) %B with GAMMA detector.

The determinations of ethanol and residual solvents (acetone and acetonitrile) were done according to the chapter < 467> of the USP (The United States Pharmacopeia (USP-40) and the National Formulary (NF-25) 2017). The Shimadzu 2010 gas chromatographic (GC) is equipped with a flame-ionization detector, a splitless injector system, and DB-WAX 30 m × 0.530 um (Agilent) column. The carrier gas was He with a flowing rate of 11 mL/min, using the temperature program. 40 °C for 2 min, then the temperature was increased at rate of 1 °C / min to 44 °C, and 20 °C / min to 200 °C to finally keep it to maintained at 200 °C for 1 min. The injection port and detector temperatures were maintained at 225 °C and 230 °C, respectively.

The gamma spectrometry was performed on a 1023-channel Ortec multichannel analyzer with 1 “× 1” Na(Tl)I crystal. Activity measurements were performed on a Capintec CRC 25 ionization chamber.

Pyrogen was tested in a Endosafe® PTS Cartridge (Charles River).

The sterility test was performed according to the chapter < 71> of the USP (The United States Pharmacopeia (USP-40) and the National Formulary (NF-25) 2017).

#### Stability in plasma and in final formulation vial

The stability in the labeling milieu, after purification and preconditioning [^18^F]-AlF-PSMA, was performed after incubation the final formulation vial at room temperature for 4 h.

The stability in human plasma of [^18^F]AlF-PSMA-11 (100 μl) was performed incubating 900 μl human plasma at 37 °C up to 4 h. After 1, 2, 3, and 4 h incubation, samples (200 μl) were precipitated with ethanol (200 μl), centrifuged (12,000 rpm, 5 min) and analyzed.

In both cases, samples were analyzed by HPLC, using previously described chromatographic conditions.

#### Animals study with tumour cell line

Nude N: NIH (S) – Foxn 1nu mice 5–7 months old were used as prostate cancer tumour model, for in vivo studies. Animals were housed in racks with filtered air under controlled conditions temperature (24 ± 1) °C and relative humidity (40–60)%). They were maintained on a 14:10 h light/dark cycles in the CUDIM animal facility with food and water ad libitum.

All protocols for animal experimentation were performed in accordance with institutional, national and international guidelines for the use of research animals, with the approval of the CUDIM Bioethics Committee’s requirements and under the current ethical regulations of the national law on animal experimentation No. 18.611 (Commission of ethics for animal studies (CEUA); National commission of experimentation with animals (CNEA)).

For the development of a xenographic human prostate cancer model, LNCaP human prostate cancer cell line (ATCC ATCC® CRL-1740™).

### Cell transplantation

A xenographic human prostate cancer model was used as the prostate cancer tumour model. 5 million LNCaP cells, suspended in 200 μL of culture media (RPMI) with matrix gel, were injected subcutaneously into the left upper leg of male nude mice (2 months old). Appropriate tumour volumes (100–350 mm^3^) were achieved between four to six weeks post inoculation. Percutaneous testorerone was daily applied to each mice. Tumours were measured once a week with a microcalliper in two dimensions, and tumour volumes were calculated as (smaller diameter)^2^ x larger diameter x π/6.

### In vivo PET/CT imaging studies

PET/CT scans were performed using a small animal tri-modality PET/SPECT/CT scanner (TriumphTM, TriFoil, Inc., US) based on Quad-APD detector modules coupled with LYSO/LGSO scintillators (spatial resolution: 1.0 mm; axial field of view (FOV): 3.75 cm).

Data were acquired in list mode in a 184 × 184 × 31 matrix with pixel size of 0.25 × 0.25 × 1.175 mm and a coincidence window width of 22.22 nsec. The animals were anesthetized with 2% isofluorane in an oxygen flow of 2 L/min, placed in prone position on the scanner bed and injected iv into the tail vein with 100–200 μL of [^18^F]AlF-PSMA-11 (33–46 MBq).

The xenograft tumours mice, images were obtained when tumours reached an optimum size. PET dynamic images acquisition started at t = 0 min after radiotracer administration and was performed over 60 min for the tumour model (1 frame × 5 min, 1 frame × 15 min, 2 frames × 20 min). CT examination was performed for 1.98 min (field of view = 4.7 cm). Mice were scanned in a random order.

Sinograms were reconstructed using 3D maximum likelihood expectation maximization (3D–MLEM) with 30 iterations.

Image processing and semi-quantitative analysis was done using PMOD software, v.3.4. (PMOD Technologies, Ltd., Zurich, Switzerland). PET studies were co-registered with the corresponding CT scan studies for anatomical localization. Images were displayed as coronal, sagittal and axial slices. Volumes of interest (VOIs) were drawn manually over the tumour and contralateral muscle for tumour bearing mice, to generate time-activity curves and calculate T/M ratios (T = Tumor, M = muscle).

## Results and discussion

An automated process is in general need for the clinical use. This fact allows the production of high activities to be used in a large number of studies. This automated process is with required for the GMP production of fluorine radiopharmaceuticals. Automation of radiopharmaceutical production is also desirable to reduce the risk for patient.

The automated synthesis of [^18^F]AlF-PSMA-11 was achieved with a radiochemical purity higher 90% and a yield of about 18%. The processes were optimized to obtain a product approved to quality control according to a radiopharmaceutical to be administered in a context of a clinical routine.

We analyzed each step of the automate synthesis in the following paragraphs.

### First step

Prior to the synthesis, it was necessary to purify the ^18^F^−^ of the target to have it in the form of [^18^F]NaF in an acetate buffer at the appropriate pH. For this purpose, a QMA column was used and the ^18^F of the target was washed with ultrapure water using the target line to perform the washing. Then the ^18^F retained in the column was eluted using 500 uL of buffer, being the smallest volume which enabled a complete elution of the QMA.

### Second step

The manual synthesis of [^18^F]AlF^+ 2^ using purified fluoride was optimized with a pH of 4.5. The pH was critically important for the formation of [AlF]^+ 2^ complex. If the pH is too high, the metals would have hydroxide complexes and precipitate on would take place. If it is too low the equilibrium would be displace towards other species. Previous studies of the AlF complexes suggested that the optimum pH was around 4, and the relation between aluminum and fluoride was around 1:1 (Martin 1988; 1986).

### Third step

**Fig. 3:**
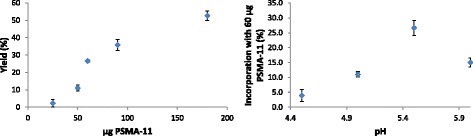
Influence of peptide mass and pH in labeling (*n* = 3)

For the labeling the PSMA-11, the peptide was added from the vial 4 together with ethanol and sodium acetate. Typically, the presence of an organic co-solvent (as ethanol) improved the efficiency of radiolabeling (Martin 1986). For its use in several patients it is necessary to work with high ^18^F activities and with larger volumes, since it is not possible to use small volumes in the automated platform (Guo et al. 2014; Allott et al. 2017). In order to obtain a high specific activity, 60 μg of peptide was used (with a concentration in the final solution of 35 μg/mL) and the influence of pH on the labeling was studied, resulting in an optimum pH of 5.5. The peptide, ethanol and 0.5 M ultrapure sodium acetate were added together to achieve the desired pH. As in the automated process higher volumes are required than in the manual labelling, not suitable yields were achieved with a mass of the peptide lower than 60 μg. We also tried with 90 and 180 μg of the peptide, increasing the yield up to 52%. As a compromise between specific activity and the cost of the peptide, 60 μg was considered the best option (see Fig. 3).

### Fourth step

During the manual optimization C18 Sep-Pak light, C18 Sep-Pak plus, Sep-Pak ligth C8 and Oasis HLB Sep-Pak plus ligth (Waters®) as extraction cartridges in solid phase were tried to purify the final product, without obtaining satisfactory results. For this reason it was decided to carry out the purification by semi preparative HPLC to achieve the final product with the highest possible radiochemical purity.

**Fig. 4:**
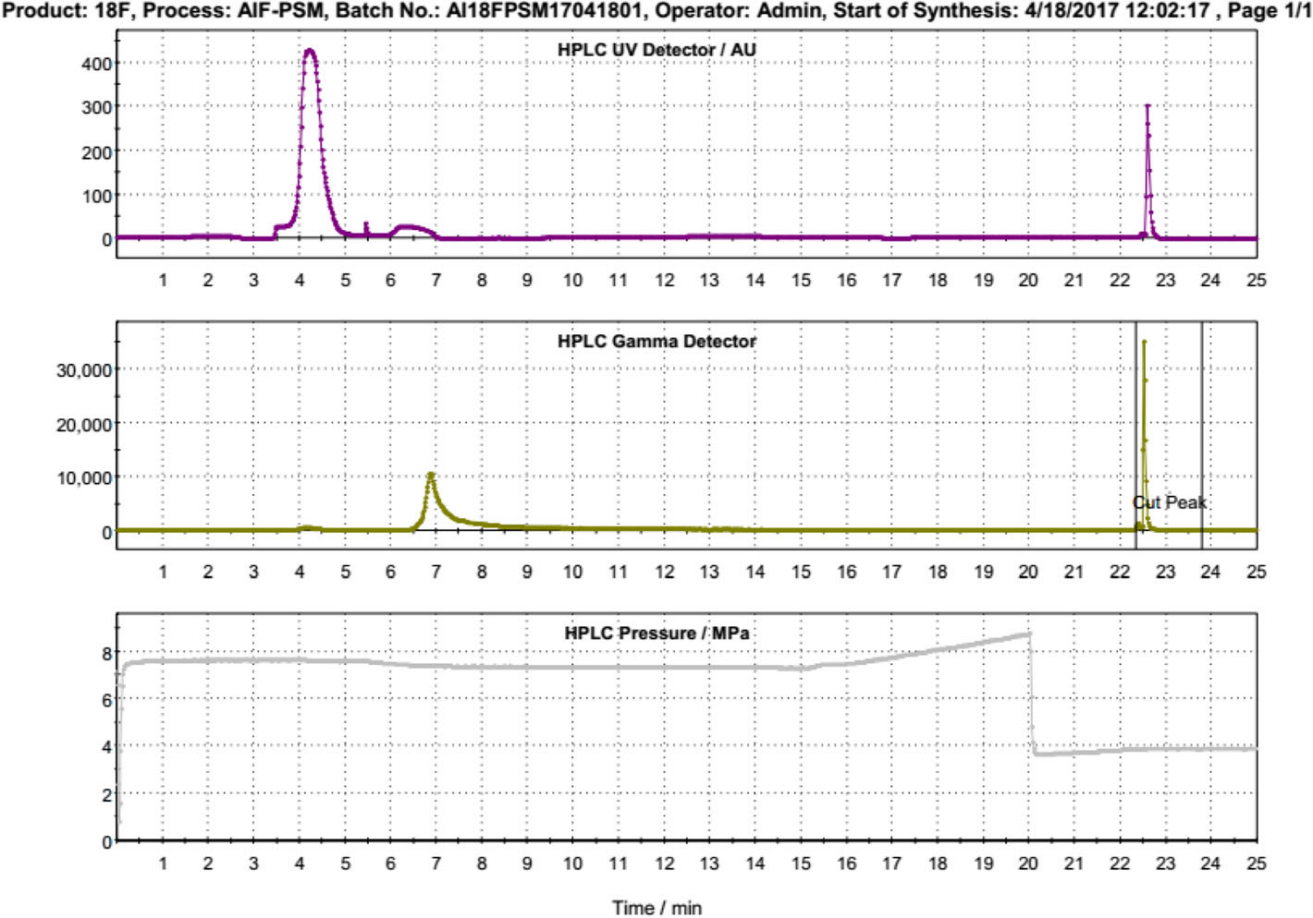
Raw HPLC profile from the FXFN module (upper: UV—280 nm, medium: gamma-ray and bottom the pressure)

A 10 mM phosphate buffer pH = 6.8 with ethanol as mobile phase were used, taking into consideration the stability of the radiopharmaceutical according to Boschi et al. (Boschi et al. 2016). The first column tested was a M-N VP 125/10 Nucleodur HTec 5 μm, with ethanol between 0 and 10%. The injection load was higher than the column capacity, which did not enable to obtain a suitable separation. Then, eventhough a M-N VP 250/10 Nucleodur C18 5um column enabled to improve the separation, a final radiochemical purity higher than 90% was not achieved. Finally, M-N VP250/16 Nucleosil 100-5C18 column was selected, using two mobile phases as it was not possible to perform a gradient. The best conditions were using 2 mobile phases with phosphate buffer: a) 2,0% to EtOH to eluate the free fluoride and the aluminum complex, b) 8,0% to EtOH to obtaining the product. The profile obtained in the product purification chromatogram is show in Fig. 4.

In order to collect the final product directly in the product vial it is necessary to connect V18 directly to V15 (see Fig. 2).

**Fig. 5:**
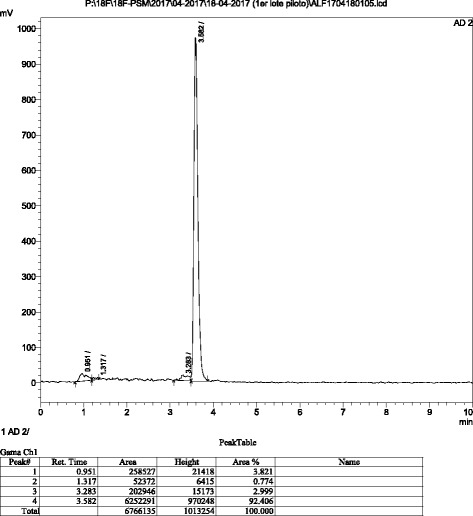
Analytical HPLC profile

The quality control verified radiochemical purity in all cases higher than 90% (see analytical chromatographic profile in Fig. 5).

The stability of the product was determined during 4 h post production (see Table 2), verifying a radiochemical purity greater than 90% in the final formulation vial, while in human plasma stability was kept in this value only during the first hour. For this reason, PET images should be acquired during this time after iv administration.

**Table 2:** Stability of the radiopharmaceutical in human plasma and in the final formulation up to 4 hs

Time (post synthesis) (hours)	Radiochemical purity in the final formulation at room temperature (%) (n = 3)	Radiochemical purity in human plasma at 37 °C (%) (n = 3)
1	94.6 ± 1.8	91.0 ± 0.8
2	92.8 ± 2.5	65.3 ± 3.2
3	93.7 ± 2.0	35.4 ± 1.9
4	94.2 ± 2.8	22.3 ± 2.6

Our results with the automated synthesis were in agreement with those previously reported by N. Malik, et by a manual process. In this paper the optimal pH of labeling turned out to be 5 and the stability was greater than 90% at 4 h post production (Malik et al. 2015).

The pilots batch were carried out under the optimized conditions, increasing activities in order to evaluate the radiopharmaceutical behavior at different activity levels, as shown in Table 3. [^18^F]AlF-PSMA was obtained with a yield of 18 ± 3% (*n* = 7), not decay corrected (NCD) starting off from 500 to 2000 mCi the 18F and with a radiochemical purity of 95 ± 3% (n = 7) and a specific activity in a range of 58 up to 544 GBq/μmol.

**Table 3:** Production and QC results of the pilot batches

	${1}^{\underline{0}}$	${2}^{\underline{0}}$	${3}^{\underline{0}}$	${4}^{\underline{0}}$	${5}^{\underline{0}}$	${6}^{\underline{0}}$	${7}^{\underline{0}}$
Production parameters							
Activity produce by cylotron(MBq)	17,700	34,400	57,500	50,600	50,600	59,200	80,000
Final Activity(MBq)	2,660	5,760	7,800	10,270	9,580	13,140	17,350
Final volume(mL)	5.80	6.16	6.09	5.13	4.20	5.71	5.75
Yield(EOS)(%)	15.0	16.8	13.6	20.3	18.9	22.2	21.7
QC parameters							
pH	7.0	7.0	7.0	7.0	7,0	7.0	7.0
Radiochemical purity(%)	92.8	100	96.8	94.0	95.1	96.2	91.7
Residual solvent	OK	OK	OK	OK	OK	OK	OK
Ethanol(%)	6.0	5.6	5.9	5.0	5.2	4.1	4.8
t_1/2_(min)	107	108	110	115	109	109	107
Pyrogen (EU/mL)	< 10	< 10	< 13.3	< 10	< 10	< 10	< 10
Sterility	sterile	sterile	sterile	sterile	sterile	sterile	sterile
Specific activity (GBq/umol)	58	180	210	357	329	412	544

The pilot batches were obtained in agreement with QC specifications. With respect to aluminum, the determination was discarded because it was used in the labeling 1.2 μg/labeling, which corresponds to 0.24 μg/mL in the final formulation. For other radiopharmaceuticals (such as ^99m^TcO_4_^−^), the USP 40 allows a limit of up to 10 μg/mL In the labeling. The aluminum is more than 40 times below the limit established for other radiopharmaceuticals.

Recently Kersemans et al. (n.d.) have reported an automated synthesis of this radiopharmaceutical in a modified SynthraFCHOL synthesis module (Synthra GmbH, Hamburg, Germany), with a similar e.o.s yield. 100 to 200 μg of the peptide were necessary to achieve a labelling yield higher than 70%. The radiopharmaceutical was stable in buffer phosphate 5 mM pH 7 at least during 150 min.

**Fig. 6:**
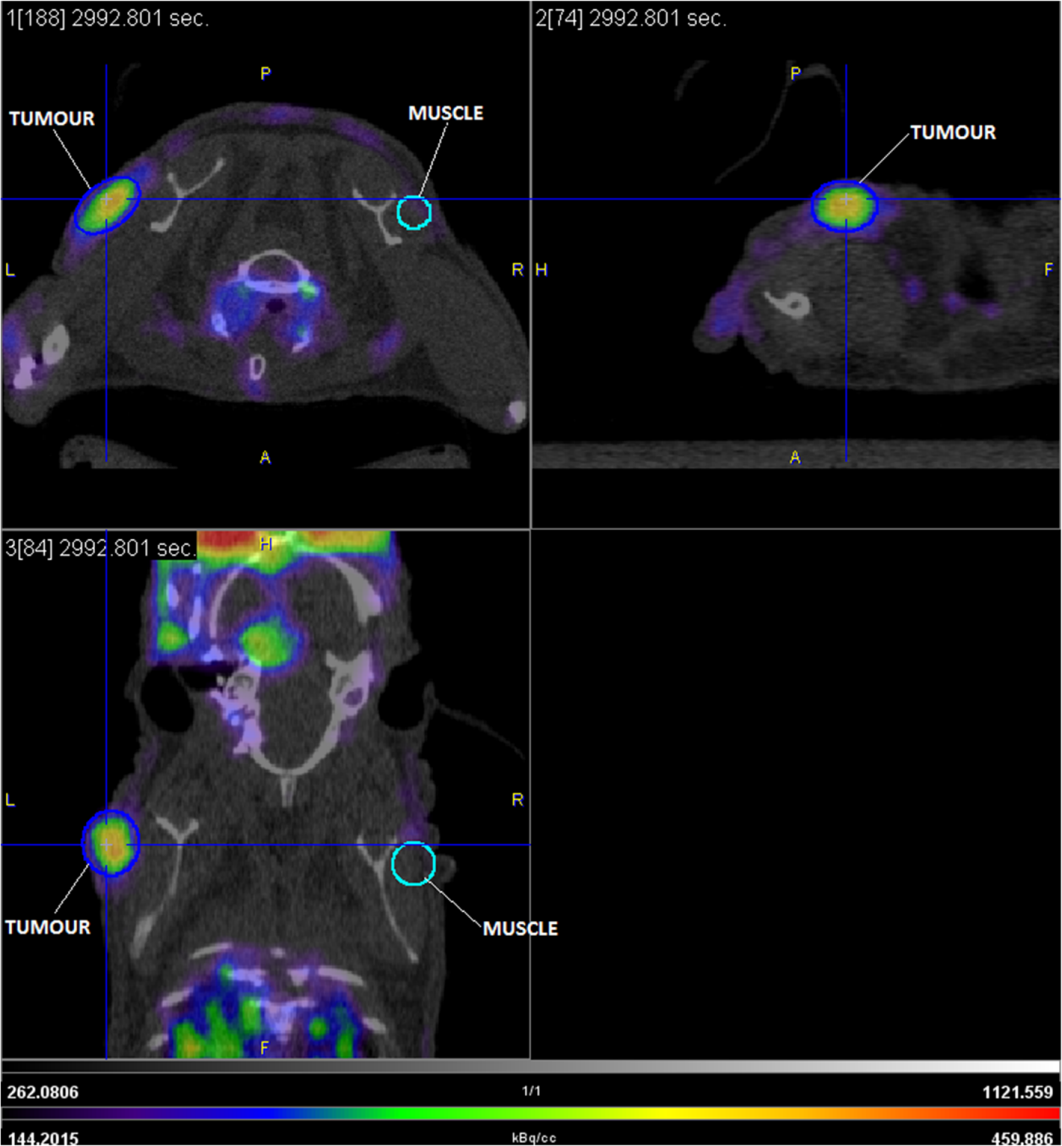
Xenografic tumor mice (LNCaP cell, Nude N: NIH (S) – Foxn 1nu mice) image acquired after administration of of [18F]ALF, using a small animal tri-modality PET/SPECT/CT scanner (TriumphTM, TriFoil, Inc., US)

PET studies showed higher tumour uptake over contralateral muscle throughout time (see Fig. 6). While the concentration of tracer in tumour increased with time, the muscle uptake decreased, providing a mean T/M ratio of 2.2 ± 0.9 at 60 min (*n* = 3).

## Conclusions

The automation of the labeling with [^18^F]AlF of different peptides presents an advantage over the traditional one with ^68^Ga, such as: (i) the coordination of a larger number of studies and, (ii) the delivery of the radiopharmaceutical to other PET clinics. Besides, the radionuclide physical properties could enable to improve the sensibility of the PET study, detecting a further number of lesions.

A suitable method for the automated synthesis of [^18^F]AlF-PSMA-11 was developed in the Ge TRACERlab FXFN platform. The proposed synthesis proved to be effective and robust, allowing the production of [^18^F]AlF-PSMA-11 with high radiochemical purity.

The labeling and purification were optimized, obtaining a product with a suitable molar activity and stability during 4 hs in the final formulation vial. This methodology enables GMP productions of [^18^F]AlF-PSMA-11 for routine use.
